# Echinochrome A Reverses Kidney Abnormality and Reduces Blood Pressure in a Rat Model of Preeclampsia

**DOI:** 10.3390/md20110722

**Published:** 2022-11-17

**Authors:** Huixing Cui, Junxian Liu, Elena A. Vasileva, Natalia P. Mishchenko, Sergey A. Fedoreyev, Valentin A. Stonik, Yinhua Zhang

**Affiliations:** 1 Department of Physiology & Biomedical Sciences, Seoul National University College of Medicine, Seoul 110-799, Republic of Korea; 2 Department of Research Center, Yanbian University Hospital, Yanji 133000, China; 3 G.B. Elyakov Pacific Institute of Bioorganic Chemistry, Far-Eastern Branch of the Russian Academy of Science, 690022 Vladivostok, Russia

**Keywords:** angiotensin II, apoptosis, blood pressure, Echinochrome A, kidney, preeclampsia, TNF–α

## Abstract

We aimed to observe the effects of Echinochrome A (Ech A) on systemic changes using a rat model of preeclampsia. The results showed that an infusion of angiotensin II (Ang II) through an osmotic pump (1 μg/kg/min) on GD 8 increased systolic and diastolic blood pressures and reduced fetal weight and placental weight. The diameters of the glomeruli were expended and glomeruli capillaries were diminished. No change was observed in the heart and liver in the Ang II group, but epithelial structures were disrupted in the uterus. Ech A treatment on GD 14 (100 μg/μL) through the jugular vein reduced systolic and diastolic blood pressures and reversed glomerulus alterations, but the fetal or placental parameters were unaffected. Ech A only partly reversed the effect on the uterus. The mRNA expression of TNF–α was increased and IL–10 and VEGF were reduced in the uterus of the Ang II group, while Ech A restored these changes. A similar trend was observed in the kidney, liver, and heart of this group. Furthermore, Bcl–2 was reduced and Bcl–2/Bax ratios were significantly reduced in the kidney and heart of the Ang II group, while Ech A reversed these changes. We suggest that Ech A modulates inflammation and apoptosis in key systemic organs in Ang II-induced rat preeclampsia and preserves kidney and uterus structures and reduces blood pressure.

## 1. Introduction

Preeclampsia (PE) is a pregnancy-specific disorder affecting 5–8% of pregnancies. Clinically, PE is characterized by hypertension and proteinuria. It is one of the leading causes of maternal and neonatal morbidity and mortality worldwide [1,2,3].

PE is a systemic disease that causes multiple organ dysfunction, and it occurs in the second half of pregnancy and is a major cause of maternal morbidity, intensive care, cesarean sections, end-organ damages and fetal complications. As the disease progresses, eclampsia, pulmonary edema, renal failure, liver failure, and HELLP syndrome (elevated liver enzymes, decreased platelets, and hemolysis) may occur. The treatment regimen that has been used clinically is the administration of magnesium sulfate for prophylaxis [4], and in patients with severe preeclampsia with severe features, hydralazine (vasodilator) or labetalol (beta-blocker) sedatives are used to control blood pressure [5,6]. Because of the potential teratogenic effects of angiotensin-converting enzyme (ACE) inhibitors and angiotensin II (Ang II) receptor blockers (ARBs) on the fetus, these drugs are limited to use during pregnancy [7]. At present, there is no effective treatment for PE, except for the termination of pregnancy, that can improve maternal and infant outcomes. Therefore, it is necessary to find an effective drug for the better control of PE.

PE is associated with immune imbalances of alternately activated immune cells and cytokines compared with normal pregnancy [8,9]. Insufficient trophoblast invasion and vascular dysregulation subsequently leads to chronic inflammation and ischemia in PE, similar to autoimmune diseases [10,11,12]. During the pathological progression of PE, pro-inflammatory T cells produce inflammatory cytokines, such as tumor necrosis factor–α (TNF–α), while anti-inflammatory cytokines that regulate immune responses such as interleukin (IL)–10 are reduced [13,14,15,16,17,18,19,20]. Such an immune imbalance worsens as the pregnancy progresses [21]. Until recently, the mechanism of this imbalance in the various organs of the mother was still unclear.

Echinochrome (Ech A) is an abundant quinone pigment of sea urchin-derived polyhydroxynaphthoquinone (EchA,7-ethyl-2,3,5,6,8-pentahydroxy-1,4-naphthoquinone) (the structure of the chemical is shown below).

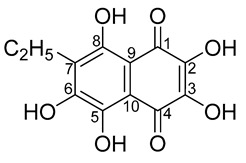



The drug is approved for medical use in Russia (PubChem CID: 135457951, C_12_H_10_O_7_) [22,23,24,25,26]. Ech A is known for its anti-inflammatory, antibacterial, and antioxidant effects [27,28,29,30,31]. In previous studies, Ech A was shown to inhibit the inflammatory effects of cytokines such as IL–1β, IL–6, TNF–α and IL–8 in cardiovascular diseases in vivo [31]. Echinochrome A (1 g) was isolated from the sea urchin *Scaphechinus mirabilis* as described previously [32] and combined with sodium carbonate (0.4 g) in a water solution that was heated until the CO_2_ was completely removed, and then it was sealed in ampoules in inert gas. The obtained solution containing 0.2 mg/mL echinochrome A was used as a stock solution.

To date, the effect of Ech A on PE had never been investigated. Therefore, we compared the effects of Ech A on pro-inflammatory cytokines and apoptosis in various organs of Ang II-induced preeclampsia rats.

## 2. Results

### 2.1. Effects of Ech A on the Ang II-Induced PE Rat Model

**Figure 1:**
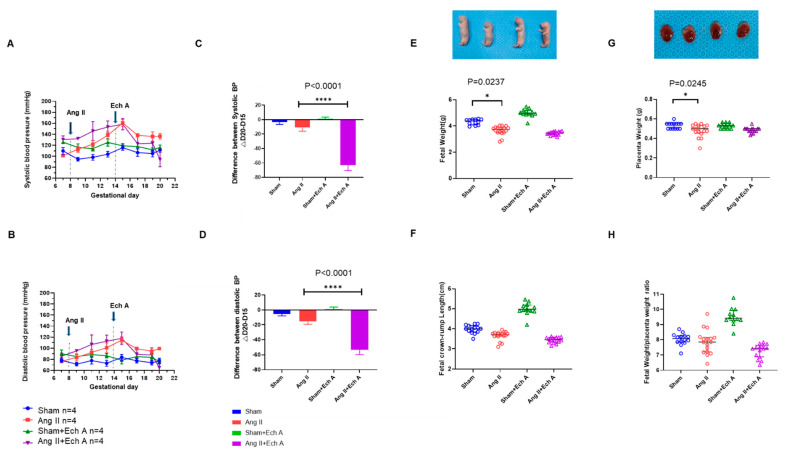
Effects of Ech A on Ang II-induced PE a rat model. (**A**,**B**) Systolic and diastolic blood pressures were increased by Ang II infusion (GD-8) in pregnant rats (the peak systolic blood pressure at GD 15 was significantly higher in the Ang II group compared to the sham group, with *p* = 0.0003 and *n* = 20 for each group). Ech A injection through jugular vein on GD 14 reduced systolic and diastolic blood pressures, and the lowest level was reached at GD 20 (*p* = 0.0008 between groups, with *n* = 20 for each group). (**C**,**D**) Blood pressure responses with Ang II and with subsequent Ech A administration were compared between the Ang II rats and the Ang II plus Ech A rats. (**E**,**F**) Image of fetal rats from the sham, Ang II, sham plus Ech A, and Ang II plus Ech A groups. As shown in E, fetal weight was reduced in the Ang II group (*p* = 0.0237 between the sham and Ang II groups, with *n* = 26 and 51, respectively), and Ech A did not restore the fetal weight in the Ang II groups (*n* = 34 and *n* = 53, respectively). (**F**) Fetal crown length was not different between the sham and Ang II groups, and Ech A did not affect fetal crown length in either group. (**G**,**H**) Images of placentas in the sham, Ang II, sham plus Ech A, and Ang II plus Ech A groups. Placental weight was reduced in the Ang II groups (*p* = 0.0245, with *n* = 26 and 51, respectively). Fetal weight/placental weight ratios were not different between the sham and Ang II groups. Ech A treatment had no effect on placental and fetal/placental weight ratios in the Ang II group (*n* = 34 and 53, respectively).

As shown in Figure 1A,B, Ang II infusion through an osmotic minipump (1 μg/kg/min) on GD 8 increased both the systolic and diastolic blood pressures from GD 11 (the blood pressure values on the highest points of GD 15 were: (1) sham: systolic blood pressure 113.2 ± 9.53, diastolic blood pressure 81.8 ± 9.0; and (2) Ang II: systolic blood pressure 153.4 ± 12.3, diastolic blood pressure 113.5 ± 10.8, with *p* < 0.0001, *p* < 0.0001, and *n* = 20 for each group).

Ech A was injected into sham and Ang II rats on GD 14 and both the systolic and diastolic blood pressures were reduced from GD 15, and the blood pressure returned to the sham level on GD 20 (systolic blood pressure: sham plus Ech A 116.6 ± 9.7, Ang II plus Ech A 92.3 ± 22.6; diastolic blood pressure: sham plus Ech A 79.0 ± 8.2, Ang II plus Ech A 61.2 ± 13.7, with *n* = 20 for each group). There were significant changes between the Ang II and Ang II plus Ech A groups on GD 20 (*p* = 0.0008). To better present the blood pressure responses to Ech A, we compared the differences in the systolic and diastolic blood pressures between the Ang II and Ang II plus Ech A groups (from GD 15 to GD 20) with their respective shams. As shown in Figure 1C,D, △D20–△D15 was significantly reduced in the Ang II plus Ech A group when compared to the Ang II group (*p* < 0.0001, Figure 1C), and similar results were observed for diastolic blood pressure (*p* < 0.0001, Figure 1D). There were no changes in the sham groups (Figure 1C,D). Therefore, Ech A reduced blood pressure in the Ang II-induced preeclamptic rats without affecting the blood pressure in the sham pregnancy rats (Figure 1A,B).

Following cesarean section, the fetal weight, fetal crown–rump length, and placenta weight were compared between the sham and Ang II groups, with and without Ech A (Figure 1E–H). The results showed that the fetal and the placental weights of the Ang II group were significantly reduced compared to those of the sham group (fetal weight: *p* = 0.0237, *n* = 26 in the sham rats and *n* = 51 in the Ang II rats; placental weight: *p* = 0.0245, *n* = 26 in the sham rats and *n* = 51 in the Ang II rats), and the fetal crown–rump length was not different between the groups. In addition, Ech A treatment did not improve the fetal and placental weights of the Ang II rats (*n* = 34 for the sham plus Ech A rats, *n* = 53 for the Ang II plus Ech A rats, Figure 1E,G).

### 2.2. H and E Staining of Maternal Kidney Cortex in the Sham and Ang II Groups with and without Ech A

**Figure 2:**
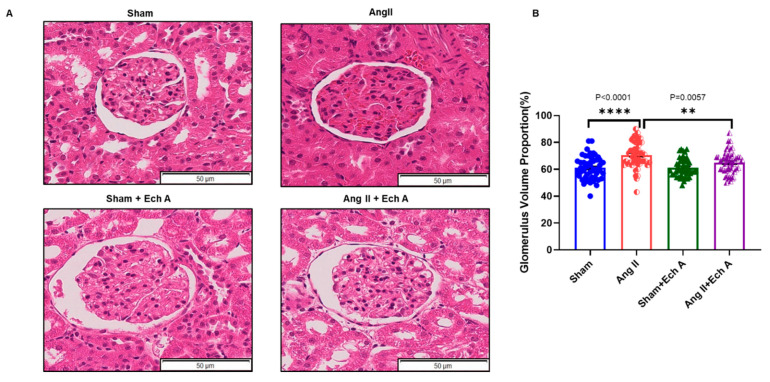
H and E staining of kidney cortex section in the sham, Ang II, sham plus Ech A, and Ang II plus Ech A groups. (**A**) Images showing the glomerular structures of the four groups. The diameters of the glomeruli in the Ang II group was increased and the capillaries were diminished in Ang II group. Ech A treatment restored the diameter changes, as well as the capillaries, in the glomeruli. (**B**) The ratios of glomeruli to renal capsules in the Ang II group were increased significantly (*p* < 0.0001, sham vs. Ang II group). Ech A treatment reduced the ratios of glomeruli to renal capsules in the Ang II group (*p* = 0.0057, Ang II vs. Ang II plus Ech A group).

Changes in the glomerulus manifests the structural and functional alterations of the kidney. H and E staining of maternal kidney cortex showed that the diameters of the glomeruli were enlarged and the glomerular capillaries were diminished in the Ang II group (Figure 2A, sham and Ang II groups). As such, the ratios of glomerular diameter and renal capsule volume were significantly larger in the Ang II group (Figure 2B, *p* < 0.0001 between the sham and Ang II groups). After Ech A treatment, the renal pathological changes were recovered, i.e., the glomerular diameters, glomerular capillaries, and the ratios of glomerular diameter and renal capsule volume were restored in the Ang II plus Ech A group (Figure 2).

**Figure 3:**
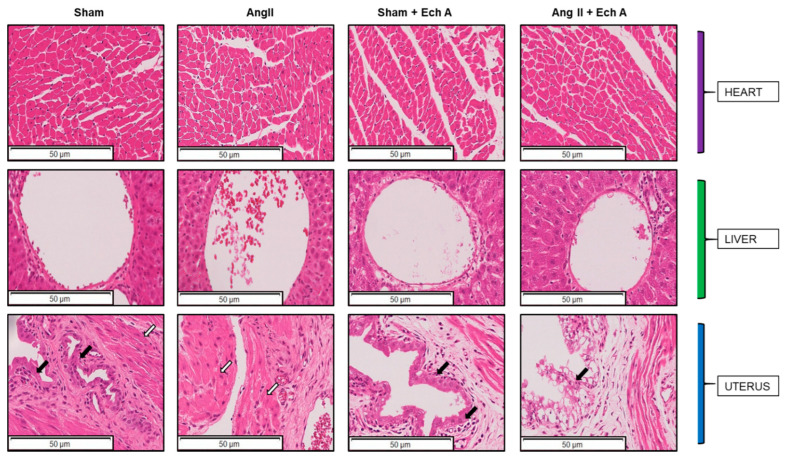
H and E staining of the heart, liver, and uterus sections of the sham, Ang II, sham plus Ech A, and Ang II plus Ech A groups. Heart: No abnormalities were observed in the structures of the myocardial fibers and Ech A did not change myocardial status. Liver: Liver sections around the portal vein area were not different among the groups. In some areas of the Ang II group, erythrocyte aggregation could be observed. Uterus: the epithelial cell structures disappeared and uterine smooth muscle tissues were not altered in the Ang II group. Ech A treatment partly restored epithelial structures, but they were surrounded by fibrin-rich tissues without changing the uterine smooth muscle structures. Black arrows indicate the locations of epithelial cells. White arrows indicate the smooth muscle tissues.

Figure 3 shows the H and E staining of the structural characteristics of the heart, liver, and uterus in the sham, Ang II, sham plus Ech A, and Ang II plus Ech A groups. The heart and liver showed no sign of changes in the Ang II group, and Ech A treatment did not affect these structures in any of the four groups. 

In the uterus, epithelial cell structures were disrupted and the smooth muscles were disoriented in the Ang II group. Ech A treatment partly restored the epithelial layers in the Ang II group, but the interstitial spaces were surrounded by fibrin-like structures (Figure 3).

### 2.3. The mRNA and Protein Expressions of TNF–α, IL–10, and VEGF in the Ang II Group and Their Responses to Ech A Treatment

**Figure 4:**
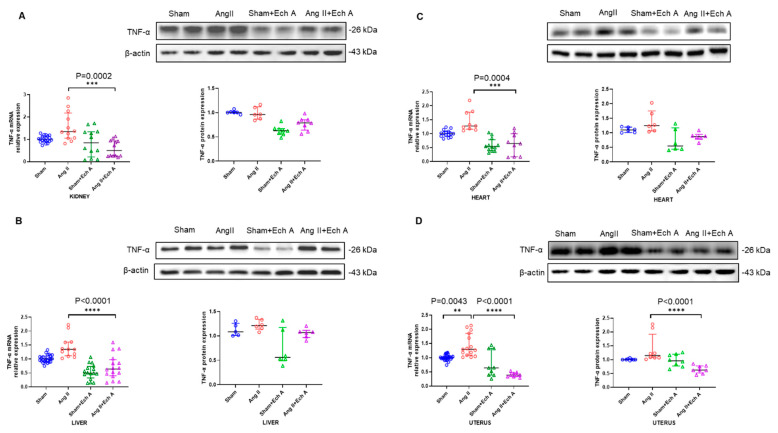
mRNA and protein expressions of TNF–α in the kidney, heart, liver, and uterus tissues of the sham, Ang II, sham plus Ech A, and Ang II plus Ech A groups. mRNA expression: TNF–α in the kidney (**A**), liver (**B**), heart (**C**), and uterus (**D**) showed an upward trend in the Ang II group, and the increment was most significant in the uterus tissues (sham vs. Ang II uterus tissues, *p* = 0.0043, with *n* = 27 vs. 15, in (**D**)). Ech A treatment significantly reduced TNF–α in all tissues of the Ang II groups (Ang II vs. Ang II plus Ech A: kidney, *p* = 0.0002, with *n* = 12 and 12; heart, *p* = 0.0004, with *n* = 9 and 9; liver, *p* < 0.0001, with *n* = 12 and 16; and uterus, *p* < 0.0001, with *n* = 15 and 9). Protein expressions: In all four tissue types, TNF–α tended to be increased in the Ang II groups. Ech A treatment decreased TNF–α expression in the kidney (**A**), liver (**B**), heart (**C**), and uterine tissues (**D**), most notably in the uterus tissues (uterus, *p* < 0.0001, with *n* = 6 and 6, in (**D**)).

Inflammation is well recognized as a core factor involved in the development of PE. We further examined inflammatory cytokines and tumor necrosis factor–α (TNF–α) in the sham, Ang II, sham plus Ech A, and Ang II plus Ech A groups. The mRNA expression of TNF–α showed an upward trend in the kidney, heart, and liver tissues of the Ang II group (Figure 4 A–C) and the levels of TNF–α were significantly increased in the uterus of the Ang II group (Figure 4D). Ech A treatment significantly decreased the TNF–α mRNA levels in the kidney, heart, liver and uterine tissues of the Ang II groups (between the Ang II and Ang II plus Ech A groups: kidney, *p* = 0.0002; heart, *p* = 0.0004; and liver and uterus, *p* < 0.0001; Figure 4). There was a trend towards increased TNF–α protein expression in all the tissues of the Ang II groups (Figure 4). Ech A treatment decreased TNF–α protein expression in the kidney, liver and heart tissues, but the response was the greatest in the uterus tissues (*p* < 0.0001, Figure 4D).

**Figure 5:**
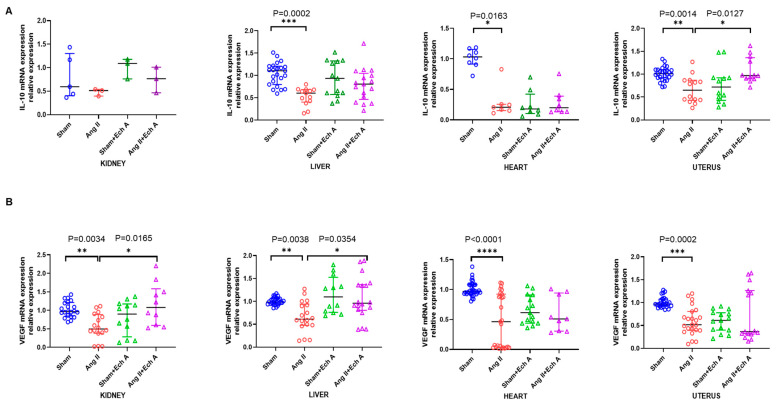
Anti-inflammatory cytokine and vascular growth factor expressions in the sham, Ang II, sham plus Ech A, and Ang II plus Ech A groups. (**A**). IL–10 mRNA expression was significantly decreased in the liver, heart, and uterine tissues of the Ang II groups (sham vs. Ang II: liver, *p* = 0.0002, with *n* = 23 and 13; heart, *p* = 0.0163, with *n* = 8 and 7; and uterus, *p* = 0.0014, with *n* = 27 and 14). Ech A significantly increased IL–10 mRNA expression in the uterine tissues of the Ang II groups (Ang II vs. Ang II plus Ech A, *p* = 0.0127, with *n* = 12 and 12). (**B**). VEGF levels were reduced in Ang II in all four types of tissues (sham vs. Ang II: kidney, *p* = 0.0034, with *n* = 21 and 16; liver, *p* = 0.0038, with *n* = 27 and 18; heart, *p* < 0.0001, with *n* = 33 and 27; and uterus, *p* < 0.0001, with *n* = 27 and 23). Ech A increased the levels of VEGF in the kidneys and livers of the Ang II groups (Ang II vs. Ang II plus Ech A: *p* = 0.0105, with *n* = 12 and 10 for the kidney tissues and *p* = 0.0354, with *n* = 12 and 18 for the liver tissues). No changes were observed in the heart and uterus tissues.

In contrast, the mRNA expression of the anti-inflammatory cytokine interleukin (IL)–10 was decreased in the hearts, livers, and uteruses of the Ang II group (heart, *p* = 0.0163; liver, *p* = 0.0002; and uterus, *p* = 0.0014; Figure 5A). The mRNA expression of (IL)–10 was significantly increased in the uterus tissues after Ech A treatment (uterus, *p* = 0.0127; Figure 5A). 

In addition, the mRNA expression of VEGF was reduced in the heart, kidney, liver, and uterus tissues in the Ang II groups (kidney, *p* = 0.0034; liver, *p* = 0.0038; heart, *p* < 0.0001; and uterus, *p* = 0.0002). Ech A treatment increased VEGF mRNA expression in the kidneys and livers of the Ang II groups (kidney, *p* = 0.0165 and liver, *p* = 0.0354; Figure 5B).

These data all suggest that Ech A exerts an anti-inflammatory capacity, especially in the uterus, and improves vascular regulation in the organs, including the kidneys, in a rat model of Ang II-induced PE.

### 2.4. Bax, Bcl–2, and Their Ratios in the Kidneys, Livers, Hearts, and Uteruses of the Four Groups with and without Ech A Treatment

**Figure 6:**
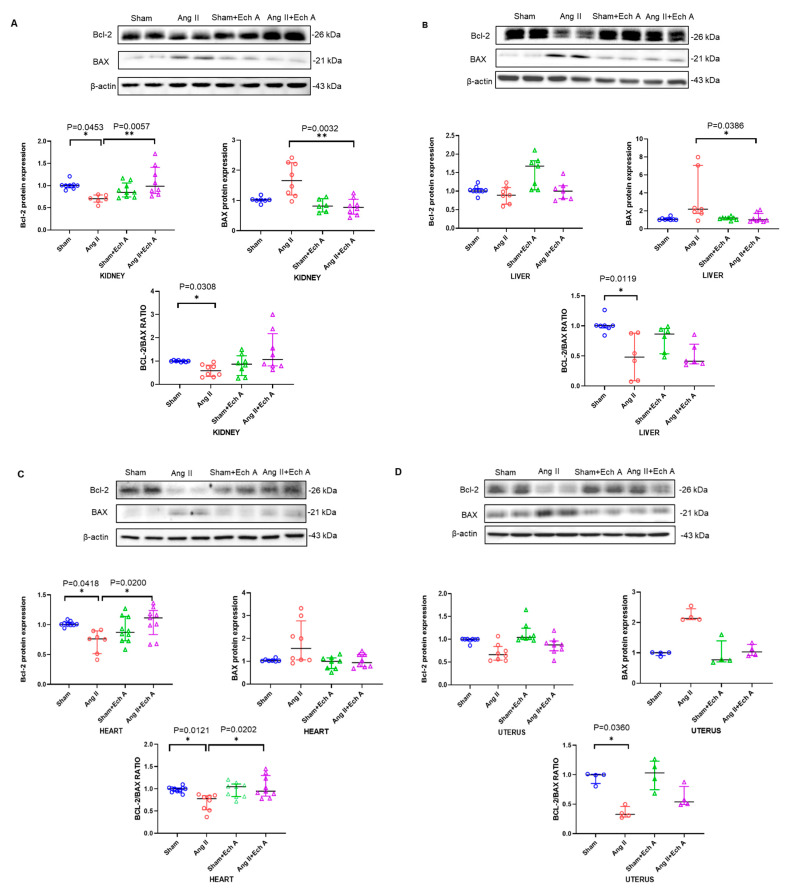
Bcl–2 and Bax protein expression and Bcl–2/Bax ratios in the kidney (**A**), liver (**B**), heart (**C**), and uterus tissues (**D**) of the sham, Ang II, sham plus Ech A, and Ang II plus Ech A groups. Bcl–2 was reduced in the kidney and heart tissues of the Ang II group, but it was not reduced in other tissues (sham and Ang II kidneys: *p* = 0.0453, with *n* = 8 and 6; and heart tissues: *p* = 0.0418, with *n* = 7 and 5). Bax was not affected in these tissues, and as such, Bcl–2/Bax ratios were reduced significantly in the hearts of the Ang II group (heart, *p* = 0.0121). Ech A treatment restored Bcl–2 changes in the hearts of the Ang II groups (Ang II and Ang II plus Ech A hearts, *p* = 0.02, with *n* = 5 and 7, in (**C**). Bcl–2/Bax ratios were increased in the heart tissues (Bcl–2/Bax between Ang II and Ang II plus Ech A hearts, *p* = 0.0202, in (**C**). There was a clear trend for Bcl–2/Bax to be increased in the kidney (in (**A**)).

Bcl–2 and Bax are two key factors that regulate apoptosis in the cells, and we tested whether Ech A may affect apoptotic status in the sham and Ang II groups. Figure 6 shows that the expression of Bcl–2 protein in the kidney and heart tissues of the Ang II group was significantly decreased (kidney, *p* = 0.0453 and heart, *p* = 0.0418; Figure 6). Bax protein expression was not affected (Figure 6). Therefore, the expression of Bcl–2/Bax ratios were decreased in the kidney (*p* = 0.0308) and heart (*p* = 0.0121). Similar reductions were observed in the livers and uteruses of the PE rats (liver, *p* = 0.0119 and uterus, *p* = 0.036). Ech A treatment significantly increased Bcl–2/Bax ratios in the Ang II-induced preeclampsia rats (heart, *p* = 0.0202; Figure 6). Similar increments were observed in the kidney, although they did not reach statistical differences (Figure 6).

The results suggest that Ech A may ameliorate maternal symptoms by regulating apoptotic status in the kidney and heart of Ang II-induced preeclampsia rats.

## 3. Discussion

Echinochrome A (Ech A) is an active pigment from sea urchin shells (a quinoid of the polyhydroxynaphthoquinone family). Ech A is known for its anti-inflammatory, antibacterial, and antioxidant effects. Preeclampsia (PE) is characterized by hypertension and proteinuria beginning at 20 weeks of gestation. The underlying abnormal factors include placental damage, incomplete spiral arterial remodeling, and endothelial damage, imbalance of immune factors, inflammatory factors, mitochondrial stress, and imbalance of pro-angiogenic and anti-angiogenic substances can lead to the development of the disease. Our results show that Ech A can effectively reduce high blood pressure and restore kidney structural damages and partly reverse the alterations in the uterus of Ang II-induced PE rats. Ech A exerted these functions through inhibiting the expression of TNF–α and increasing the expression of IL–10 in the uterine tissues of Ang II-induced PE rats. Ech A may reduce apoptosis in the kidney and heart, restore the VEGF of kidney and liver, and improve endothelial function. It is suggested that pro-inflammatory factors have anti-inflammatory ability in our Ang II-induced PE rat model.

Ech A is a drug which was approved by the Ministry of Health of the Russian Federation for its clinical application [33,34,35]. Ech A has been used in the treatment of inflammation of the heart or eye disease [36,37]. PE is well known to be associated with chronic inflammation and is manifested by increases in pro-inflammatory cytokines, such as TNF–α, which contributes to the exacerbation of the pathogenesis of PE. Decreases in anti-inflammatory cytokines, such as IL–10 from Treg, have been associated with PE [38,39,40], and they are responsible for the increment of inflammatory cytokines, TNF–α, IL–6 and IL–17, from Th 1 [41,42]. The pro- and anti-inflammatory cytokine imbalance can lead to chronic peripheral and placental inflammation, featured by endothelial dysfunction or increased permeability of the systemic or placental vasculature, which further complicates the patho-progression of an abnormal pregnancy. In addition, excess TNF–α in PE can specifically inhibit trophoblast migration and integration [43,44,45]. Therefore, management of the inflammatory response is recommended to facilitate the treatment of PE. In line with previous studies, our results clearly showed increased mRNA and proteins of TNF–α in various tissues of PE rats (e.g., uterus tissue), which was reversed by Ech A treatment. This is the first study showing the favorable effects of Ech A in an animal model of PE.

PE is secondary to abnormal placenta and placental vascularization and reduced placental blood flow and chronic hypoxia are the main triggers for placental trophoblast and endothelial apoptosis. The cell or organelle debris (including mitochondrial DNA (mtDNA) activates a series of signaling pathways in the system to induce inflammation and cardiovascular dysfunction and damage. Therefore, placental apoptosis has long been considered a major cause of the organ damage (including maternal organ cell apoptosis) of PE. Bcl–2 and Bax proteins are members of the Bcl–2 family and have opposing functions to regulate apoptosis. As an anti-apoptotic protein, the increase in Bcl–2 usually indicates that the anti-apoptotic function of the cells is enhanced, and the increase in Bax protein expression indicates the promotion of apoptosis. Therefore, changes in Bcl–2 and Bax can directly reflect the level of apoptosis in cells. Our results showed that Ech A increased the expression of Bcl–2 and decreased the expression of Bax in the placentas of preeclamptic rats. It is suggested that the regulation of apoptosis gene expression by Ech A may also be involved in ameliorating the maternal symptoms of Ang II-induced PE.

Of note, Ech A treatment did not affect fetal or placental weights in the Ang II group. However, Ech A significantly increased fetal weights and improved the ratios of fetal/placental weights in the sham group, indicating that even in a normal pregnancy, Ech A may be beneficial for fetal development. Indeed, the mRNA and protein expressions of TNF–α were reduced in the kidney, heart, liver, and uterus of the rats. IL–10 was not affected. Furthermore, Ech A induced structural changes in the uterus of the sham rats (fibrin-like tissue accumulations in the interstitial spaces, Figure 3), and this was accompanied by a significant reduction of VEGF in these tissues following Ech A treatment (Figure 5B). These results suggest that Ech A possesses the potential to be a natural supplement for fetal development in normal pregnancy. The efficacy and safety of Ech A supplementation warrants further investigation with wider ranges of pregnancy models, including human, as well as the recovery of uterus structures after successful deliveries.

Collectively, our results clearly demonstrated that Ech A administration prevents tissue damage in an Ang II-induced PE rat model by modulating tissue inflammation and apoptosis. The effects of Ech A on maternal systemic organs and fetal development in normal pregnancies need thorough investigation.

## 4. Materials and Methods

### 4.1. Preeclampsia Rat Model

Animal experimental protocols were performed in accordance with the Guide for the Care and Use of Laboratory Animals published by the US National Institutes of Health (NIH, Publication No. 85-23, revised 196), and approved by the Institutional Animal Care and Use Committee of the Laboratory Animal Center, Seoul National University, South Korea [SNU-190413-5-3]. A total of 16 Sprague-Dawley female pregnant rats were used in the study. The duration of pregnancy is shown as the gestation day (GD). Pregnant rats (GD 7, weighing 230–270 g) were provided by the KOATECH Company (Korea). The rats were randomly divided into 4 groups: sham, Ang II, sham plus Ech A, and Ang II plus Ech A. On GD 8, Ang II was infused into the pregnant rats through an osmotic minipump (1 μg/kg/min) and Ang II was applied until GD 20. Echinochrome A (100 μg/μL) was injected into the jugular veins of the pregnant rats in the sham and Ang II groups on GD 14. The rats were sacrificed on GD 20 and the heart, kidney, liver, and uterine tissues were collected on GD 20.

### 4.2. Measurement of Blood Pressure

The blood pressures of the pregnant rats were measured on GD 7, 9, 11, 13, 15, 17, 19, and 20 using a Noninvasive Blood Pressure Measurement Device (CODA). Individual pregnant rats were fixed and warmed to 32–35 °C for 15 min before each measurement, and 3 cycles were averaged for data analysis.

### 4.3. H and E Staining

After the cesarean procedures, organ tissues were removed, chopped into small pieces (~2 mm × 2 mm), and rinsed in 4 °C PBS solution a few times to wash off the blood. We divided the tissues into aliquots of equal sizes, and two aliquots were fixed in formalin for H and E and the others were snap-frozen in liquid nitrogen and stored at −80° for the WB and qRT-PCR experiments. Three repeats were performed on the same samples. The number of samples were added in the text.

We collected the hearts, kidneys, livers, and uteruses of the four groups on GD 20. The tissues were fixed in 4% paraformaldehyde and then transferred to a 70% ethanol solution for 24 h at 20–22 °C. The samples were embedded in paraffin and cut into 4 μm sections. Sections were stained with hematoxylin and eosin (H and E) and toluidine blue. The stained slides were visualized using Aperio ImageScope software (12.3 version; Copyright © 2022–2017 Leica Biosystems Imaging, Inc. Aperio is a trademark of the Leica Biosystems group of companies in the USA and optionally in other countries).

### 4.4. RNA Preparation and Quantitative Real-Time PCR

Total RNA was extracted from the collected tissues (heart, liver, kidney, and uterus) using Qiazol Lysis Reagent (Cat.no.79306, QIAGEN). The purified RNA concentration was determined with a NanoDrop (ThermoFisher). The primers were: TNF–α (forward, 5′-CCAGGAGAAAGTCAGCCTCCT-3′, reverse, 5′-TCATACCAGGGCTTGAGCTCA-3′), IL–10 (forward, 5′-GCTGGACAACATACTGCTGA-3′, reverse, 5′-TGCTCCTTGATTTCTGGG-3′), VEGF (forward, 5′-GCCCATGAAGTGGTGAAGTT-3′, reverse, 5′-ACTCCAGGGCTTCATCATTG-3′), and 18sRNA (forward, 5′-CGCGGTTCTATTTTGTTGGT-3′, reverse, 5′-AGTCGGCATCGTTTATGGTC).

The cDNA was amplified with SYBR green (BIONEER 2XGreenStarqPCRMaster MIX) using a real-time PCR system (Applied Biosystems 7500 Real-time PCR) under the following conditions: 95 °C for 3 min, 40 cycles at 95 °C for 10 s, 55 °C for 3 s, 95 °C for 10 s, and 65 °C for 5 s. The specificity of the amplification products was assessed by a melting curve analysis. Relative gene expression was calculated using the comparative-threshold (Ct) method (2^−△△Ct^).

The information of the primer sequences is as follows:
TNF–α-RATCCAGGAGAAAGTCAGCCTCCTFTNF–α-RATTCAT ACCAGGGCTTGAGCTCARIL–10-RATGCTGGACAACATACTGCTGAFIL–10-RATTGCTCCTTGAT TTCTGGGRVEGF-RATGCCCATGAAGTGGTGAAGTTFVEGF-RATACTCCAGGGCTTCATCATTGR18sRNA-RATCGCGGTTCTATTTTGTTGGTF18sRNA-RATAGTCGGCATCGTTTATGGTCR

### 4.5. Western Blotting Analysis

Proteins were extracted from the heart, liver, kidney, and uterus tissues in lysis buffer containing 150 mM NaCl, 50 mM Tris-HCl, 1 mM EDTA, and 1% Triton X-100 with a phosphatase inhibitor cocktail (Roche, Basel, Switzerland) at pH 7.4. The lysates were boiled at 95 °C for 10 min and then fractionated by SDS/PAGE and transferred to PVDF membranes in 25 mM Tris, 192 mM glycine, and 0.01% SDS, 20% methanol. The membranes were blocked in 1X TBS containing 1% Tween-20 and 5% BSA (blocking solution) for 1 h at RT with gentle rocking. The membranes were then incubated overnight at 4 °C with the primary antibodies (BCl-2 and Bax antibody: Santa Cruz Biotechnology, Dallas, TX, USA, 1:1000; TNF-α antibody: Santa Cruz Biotechnology, 1:1000; β-actin antibody: Santa Cruz Biotechnology, 1:5000), followed by the secondary antibodies. The chemiluminescence reagent ECL was purchased from Amersham Company (Amersham, UK).

### 4.6. Statistical Analysis

Normality tests were conducted on all the data sets using the D’Agostin and Pearson method from Prism Graphpad 8.0. A *p* of >0.05 was defined as having passed the normality test, and the data are presented as means ± SDs, followed by one-way ANOVA tests for group comparisons. The data of non-normal distribution, scatterplots, and medians with interquartile ranges are presented. Nonparametric tests (Kruskal–Wallis test multiple comparisons) were performed on the latter. A *p* of <0.05 was considered significant.

## 5. Conclusions

Ech A administration in vivo during pregnancy and during the onset of PE has been shown to reduce systolic and diastolic blood pressures and reverse the deformation of renal structures (glomeruli swelling and capillary changes) through modulating inflammatory cytokine levels in the key organs of pregnant rats. Such a modulation may attenuate tissue apoptosis and significantly improve maternal burden. Ech A exhibited favorable effects on fetal development in the sham rats. The study demonstrates a proof of principle for Ech A development as a useful supplement for better pregnancy management.

## Data Availability

The data that support the findings of this study are available on request from the first and corresponding authors (H.C. and Y.Z.).
